# Downregulated exosome-associated gene FGF9 as a novel diagnostic and prognostic target for ovarian cancer and its underlying roles in immune regulation

**DOI:** 10.18632/aging.203905

**Published:** 2022-02-21

**Authors:** Zhijie Xu, Yuan Cai, Wei Liu, Fanhua Kang, Qingchun He, Qianhui Hong, Wenqin Zhang, Jianbo Li, Yuanliang Yan, Jinwu Peng

**Affiliations:** 1 Department of Pathology, Xiangya Hospital, Central South University, Changsha 410008, Hunan, China; 2 Department of Pathology, Xiangya Changde Hospital, Changde 415000, Hunan, China; 3 National Clinical Research Center for Geriatric Disorders, Xiangya Hospital, Central South University, Changsha 410008, Hunan, China; 4 Department of Orthopedic Surgery, The Second Hospital University of South China, Hengyang 421001, Hunan, China; 5 Department of Emergency, Xiangya Hospital, Central South University, Changsha 410008, Hunan, China; 6 Department of Emergency, Xiangya Changde Hospital, Changde 415000, Hunan, China; 7 Department of Pharmacy, Xiangya Hospital, Central South University, Changsha 410008, Hunan, China

**Keywords:** ovarian cancer, exosome, FGF9, prognosis, immune regulation

## Abstract

Exosome has been demonstrated to be secreted from cells and seized by targeted cells. Exosome could transmit signals and exert biological functions in cancer progression. Nevertheless, the underlying mechanisms of exosome in ovarian cancer (OC) have not been fully explored. In this study, we wanted to explore whether Fibroblast growth factor 9 (FGF9), as an exosome-associated gene, was importantly essential in OC progression and prognosis. Firstly, comprehensive bioinformatics platforms were applied to find that FGF9 expression was lower in OC tissues compared to normal ovarian tissues. Meanwhile, downregulated FGF9 displayed favorable prognostic values in OC patients. The gene enrichment of biological functions indicated that abnormally expressed FGF9 could be involved in the OC-related immune signatures, such as immunoinhibitors and chemokine receptors. Taken together, these findings could provide a novel insight into the significance of FGF9 in OC progress and supply a new destination of FGF9-related immunotherapy in clinical treatment.

## INTRODUCTION

The incidence rate of ovarian cancer ranks the third place all over the world. And of all the malignant tumors, the fatality rate of ovarian cancer is the highest [1]. In recent days, numerous kinds of clinical treatments have been applied to the patients with ovarian cancer, including surgery, chemotherapy. Whereas, for patients that are diagnosed in the late stage, the 5-year survival rate accounts for <25%. And the effects of conventional treatments are not as good as before [2]. Therefore, it is significantly important to explore a novel biomarker and new clinical therapies to improve the overall rate and prognosis of ovarian cancer patients.

Exploring the mechanisms of tumor immune microenvironment (TIME) could be beneficial for the guidance of the immune responses of cancer and predict the therapeutic molecules [3, 4]. To have a better understanding of the TIME in ovarian cancer is necessary. Because immune cells could enhance anticancer activities and decrease the recurrence rate of cancer. Immune systems combined with signaling biomarkers could play a crucial role in the prognostic prediction of ovarian cancer patients [5]. Nevertheless, more profound scientific researches regarding the relationships between TIME and ovarian cancer are still essential and needed to be fully investigated.

Exosomes were demonstrated to be normal nanovesicles which were composed of various molecules, including lipid and nucleic acids. They were found in the exocytosis of cells and could contribute to the biological functions of cells, which could serve as a biomarker in the pathological process of human diseases and cancers [6–8]. Exosomes had a strong relationship with immune responses in numerous diseases, such as cardiovascular, central nervous system and cancer. Furthermore, the utility of exosome-related immune regulation could do good to the future therapeutic progression [9, 10]. Fibroblast growth factor 9 (FGF9), as an exosome-associated gene, was first found in human glioma cells, and it has been reported to participate in the regulation in glia of central nervous system [11]. Recently, numerous studies have demonstrated that FGF9 plays a crucial part in the tumor progression. The upregulated level of FGF9 exerted great effects in the transdifferentiation of small cell lung cancer (SCLC). *In vivo* studies have identified that FGF9 induced the malignant transformation via triggering FGFR pathway [12]. Moreover, abnormally expressed FGF9 was involved in the modulation of OC invasiveness [13]. Whereas, more studies are still required to investigate the relationship between FGF9 expression and the prognosis of OC patients.

In this paper, we would explore the underlying mechanisms of FGF9 in ovarian cancer. Through comprehensive bioinformatic analysis, the expression level of FGF9 was discovered to be downregulated in ovarian cancer tissues. And the high expression of FGF9 has strong correlation with good prognosis. These findings indicated that FGF9 could be a novel prognostic prediction and immune-associated biomarker for ovarian cancer.

## MATERIALS AND METHODS

### Data acquisition

Gene Expression Omnibus (GEO) database [14] was applied to explore and download the two OC datasets, including GSE26712 [15, 16], GSE18520 [17] (Table 1). Then, we have analyzed the differently expressed genes (DEGs) between the normal ovarian tissues and OC tissues. The cut-off value was established: *p*-value <0.05 and |logFC| ≥2.0. In order to explore the co-differentially expressed genes (co-DEGs) of the exosome-associated gene dataset and two GEO datasets, the Venn plots were employed. Moreover, the Cancer Genome Atlas (TCGA) database [18] was used to obtain the expression levels and clinical statistics of OC patients.

**Table 1:** The features of two GEO datasets about gene expression profiling by array.

**GEO datasets**	**Platform**	**Sample size**		**DEGs**	**References**
		**cancer**	**normal**		
GSE26712	GPL96	185	10	82 up-regulated genes and 231 down-regulated genes	[15, 16]
GSE18520	GPL570	53	10	493 up-regulated genes and 599 down-regulated genes	[17]

Abbreviations: GEO: Gene Expression Omnibus datasets; DEGs: differentially expressed genes.

### Bioinformatics platforms

The comprehensive evaluations of differently expressed genes were downloaded from some bioinformatic platforms (Table 2). The prognostic values were evaluated by means of the Kaplan-Meier plotter [19]. This database was used for the exploration of the overall survival (OS), first-progression survival (FPS) and post progression survival (PPS) of co-DEGs in OC. Additionally, the TNMplot [20], TCGA database and GEPIA2.0 [21] further investigated the expression level of FGF9 in tumor group and normal group. Subsequently, the LinkedOmics platform [22] was used to analyze the correlation between FGF9 and co-expressed genes. At the same time, through this database, we have identified the Gene Ontology (GO) and Kyoto Encyclopedia of Genes and Genomes (KEGG) pathways. Concerning the research of the link between FGF9 and immune response and regulation, we employed the single-sample GSEA (ssGSEA), TISIDB [23] and TIMER [24]. Subsequently, the relationships between FGF9 expression and immune checkpoints, including CTLA4 and VSIR have been figured out.

**Table 2:** Bioinformatics platforms that are employed to analyze the role of FGF9 in ovarian cancer.

**Database**	**URL**	**References**
GEO	https://www.ncbi.nlm.nih.gov/gds/?term=	[14]
TCGA	https://portal.gdc.cancer.gov/	[18]
Kaplan-Meier Plotter	http://kmplot.com/analysis/	[19]
TNMplot	http://www.tnmplot.com	[20]
GEPIA2.0	http://gepia.cancer-pku.cn/	[21]
LinkedOmics	http://www.linkedomics.org/admin.php	[22]
TISIDB	http://cis.hku.hk/TISIDB/	[23]
TIMER	https://cistrome.shinyapps.io/timer/	[24]

### Statistical analysis

The findings in this study were depicted as mean ± standard deviation (SD). The difference between the normal group and the tumor group was investigated by *t*-test. *P* < 0.05 was regarded to be statistically significant.

## RESULTS

### Differently expressed genes between ovarian cancer group and normal group

**Figure 1:**
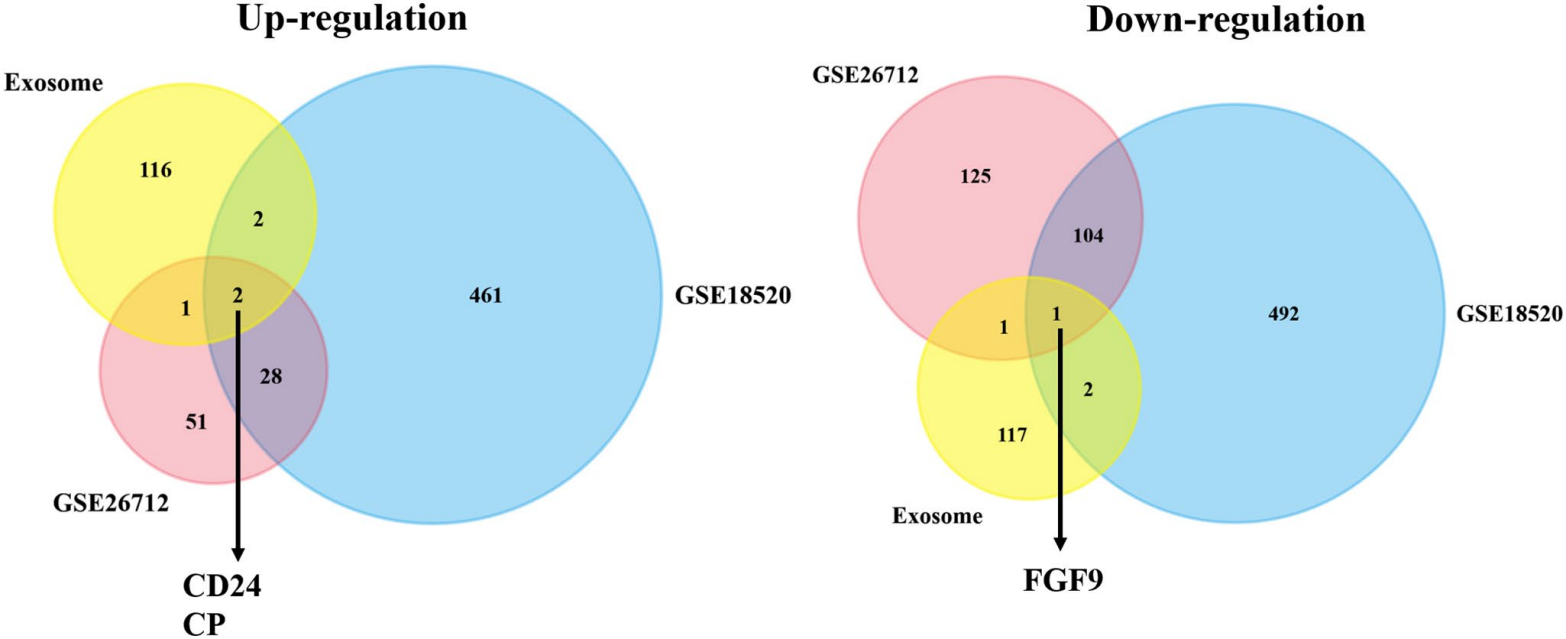
**The co-DEGs between the exosome-associated genes and two OC datasets.** The Venn plot showed that two upregulated exosome-correlated genes (CD24 and CP) and one downregulated exosome-correlated gene (FGF9) might play pivotal roles in OC progression.

Through the exploration in GEO database, we finally picked out two suitable datasets and then attained the statistics of the two datasets. The cut-off value was set up: *p*-value <0.05 and |logFC| ≥2.0. After this procedure, DEGs between ovarian cancer group and normal group were generated. And we found that in GSE26712, there were 82 genes up-regulated and 231 genes down-regulated. Meanwhile, there were 493 up-regulated genes and 599 down-regulated genes in GSE18520 (Supplementary Table 1). Besides, we applied the Venn plot (http://bioinformatics.psb.ugent.be/webtools/Venn/) to implicate the significance of exosome-correlated genes in the development of OC patients. And the Venn plot showed that two upregulated exosome-correlated genes (CD24 and CP) and one downregulated exosome-correlated gene (FGF9) might play pivotal roles in OC progression (Figure 1).

### The prognostic prediction value of FGF9 in ovarian cancer patients

**Figure 2:**
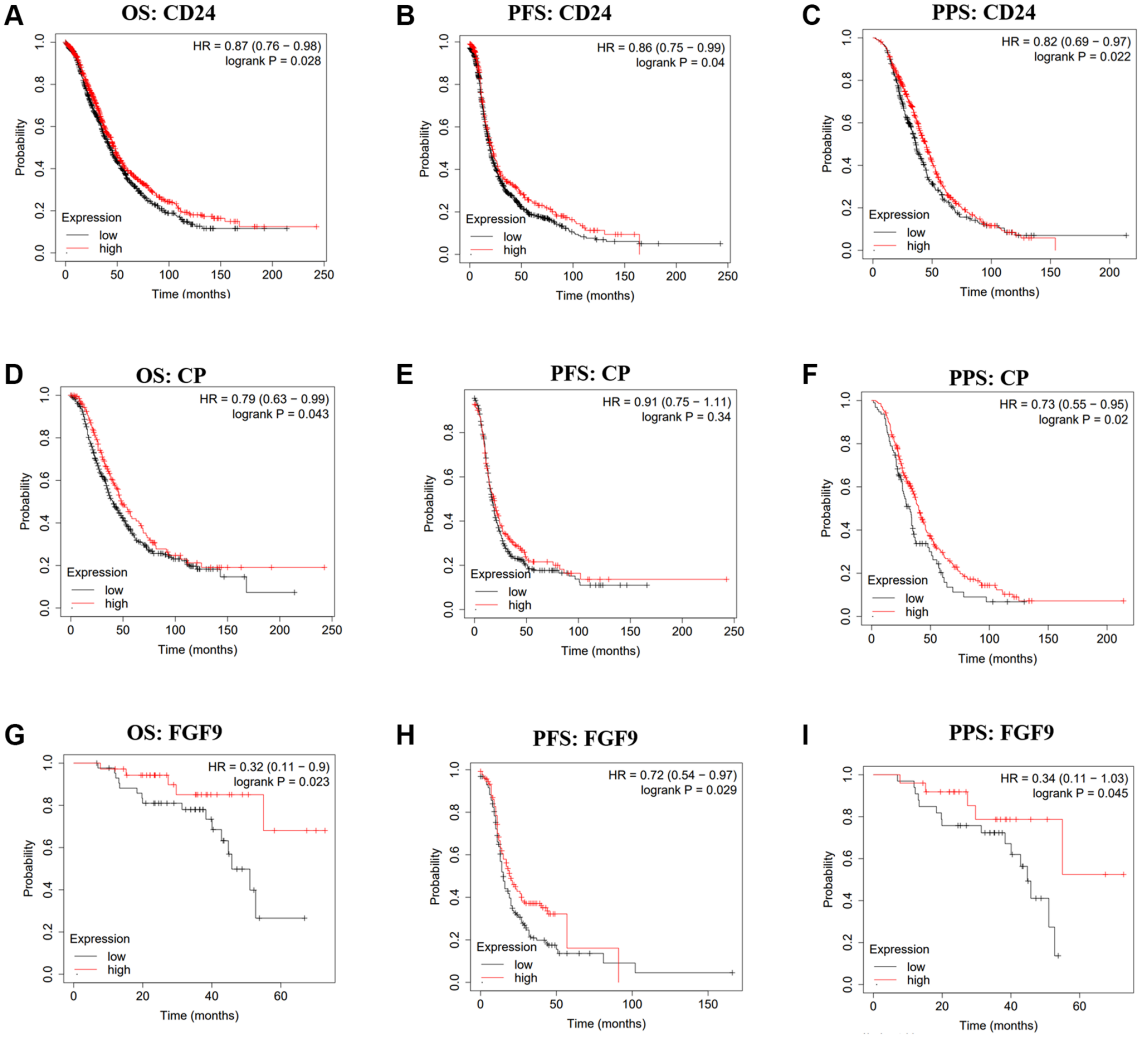
**The prognostic values of CD24, CP and FGF9 in OC.** (**A**–**I**) The prognostic values of CD24, CP and FGF9 in ovarian cancer patients. Abbreviations: OS: overall survival; PFS: progression-free survival; PPS: post progression survival.

To investigate the correlation between the expression of CD24, CP, FGF9 and the OC patients’ prognosis, the Kaplan-Meier plotter was applied. The findings have conveyed that high expression level of CD24 was linked to good OS (HR = 0.87, 95% CI = 0.76–0.98, *p* = 0.028), PFS (HR = 0.86, 95% CI = 0.75–0.99, *p* = 0.04), PPS (HR = 0.82, 95% CI = 0.69–0.97, *p* = 0.022) (Figure 2A–2C). Furthermore, high expression of CP was related to favorable OS (HR = 0.79, 95% CI = 0.63–0.99, *p* = 0.043), PPS (HR = 0.73, 95% CI = 0.55–0.95, *p* = 0.02) (Figure 2D, 2F). However, CP expression was not correlated with the PFS of OC patients (*p* > 0.05) (Figure 2E). And from the GSE14764, we could conclude that higher expression of FGF9 was linked to the better prognosis of OS (HR = 0.32, 95% CI = 0.11–0.9, *p* = 0.023), PPS (HR = 0.34, 95% CI = 0.11–1.03, *p* = 0.045) (Figure 2G, 2I). Additionally, OC patients with high level of FGF9 showed favorable PFS (HR = 0.72, 95% CI = 0.54–0.97, *p* = 0.029) in GSE9891 (Figure 2H). From these results, we speculated that FGF9 could possess the potential ability to be a prognostic biomarker.

### Downregulated expression of FGF9 in ovarian cancer group

**Figure 3:**
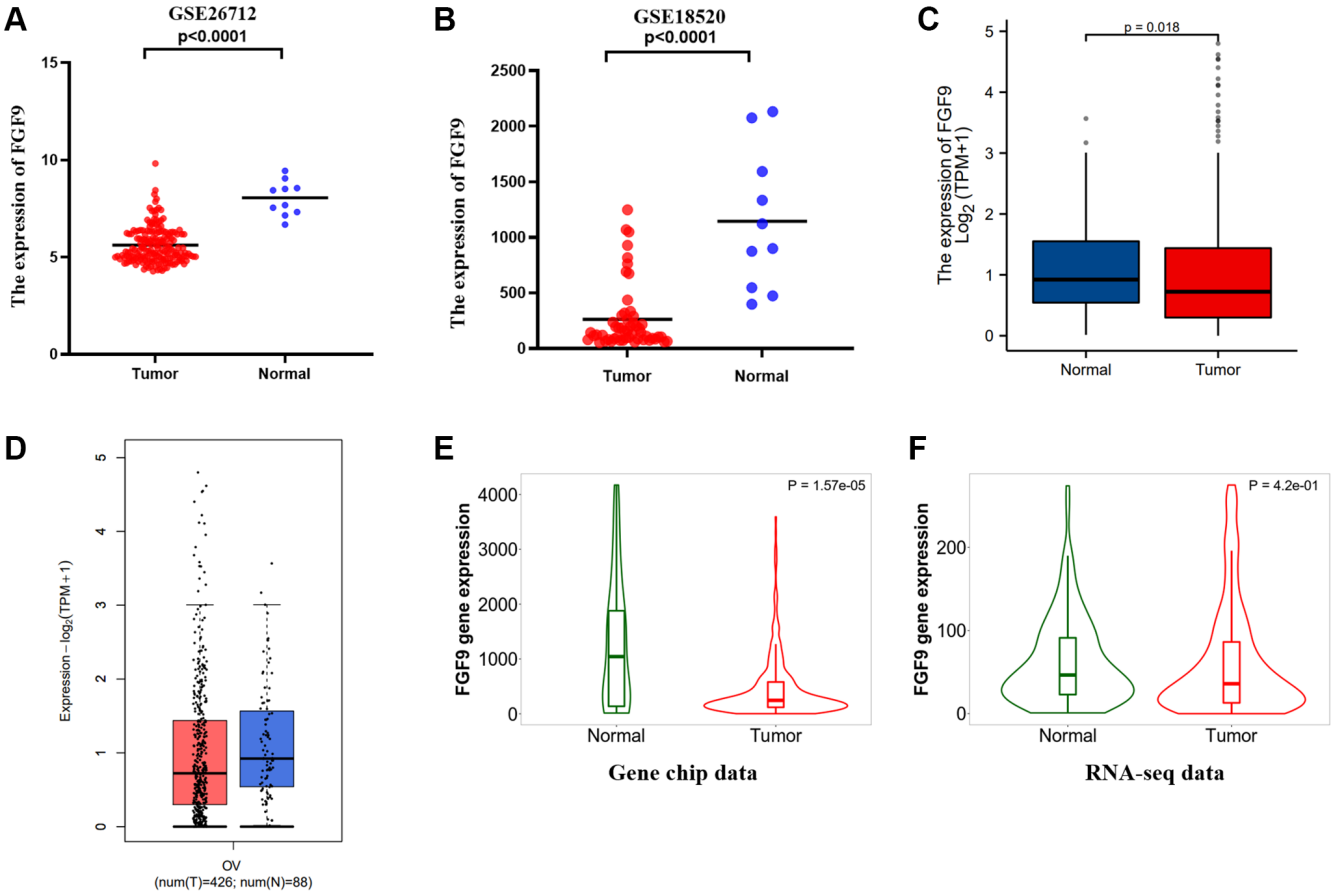
**FGF9 was down-regulated in OC patients.** (**A**, **B**) In the two datasets, the expression level of FGF9 was lower in OC tissues than that in normal ovarian tissues. (**C**, **D**) The GEPIA2.0 database and TCGA database have depicted that the expression of FGF9 decreased in OC tissues compared to normal ovarian tissues. (**E**, **F**) TNMplot database depicting FGF9 expression was lower in OC tissues compared to normal tissues from gene chip data and RNA-seq data.

Through the evaluation of the two datasets downloaded from the GEO database, we found that FGF9 expressed more highly in normal ovarian tissues than ovarian cancer tissues (*p* < 0.0001) (Figure 3A, 3B). In addition, TCGA database has verified that the expression level of FGF9 was different between the normal group and the OC group (*p* = 0.018) (Figure 3C). Meanwhile, GEPIA2.0 platform has identified that the expression level of FGF9 was higher in the normal group than that in OC group (Figure 3D). What’s more, by means of the TNMplot platform, we could know about that FGF9 mRNA expression were both lower in OC tissues from gene chip data (*p* = 1.57e-05) and RNA-seq data (*p* = 4.2e-01) (Figure 3E, 3F). The above findings have conveyed that the expression of FGF9 quietly diminished in OC group, implicating that FGF9 might be a promising inhibitor in OC patients’ progression and development.

### The co-expression network of FGF9 in ovarian cancer

**Figure 4:**
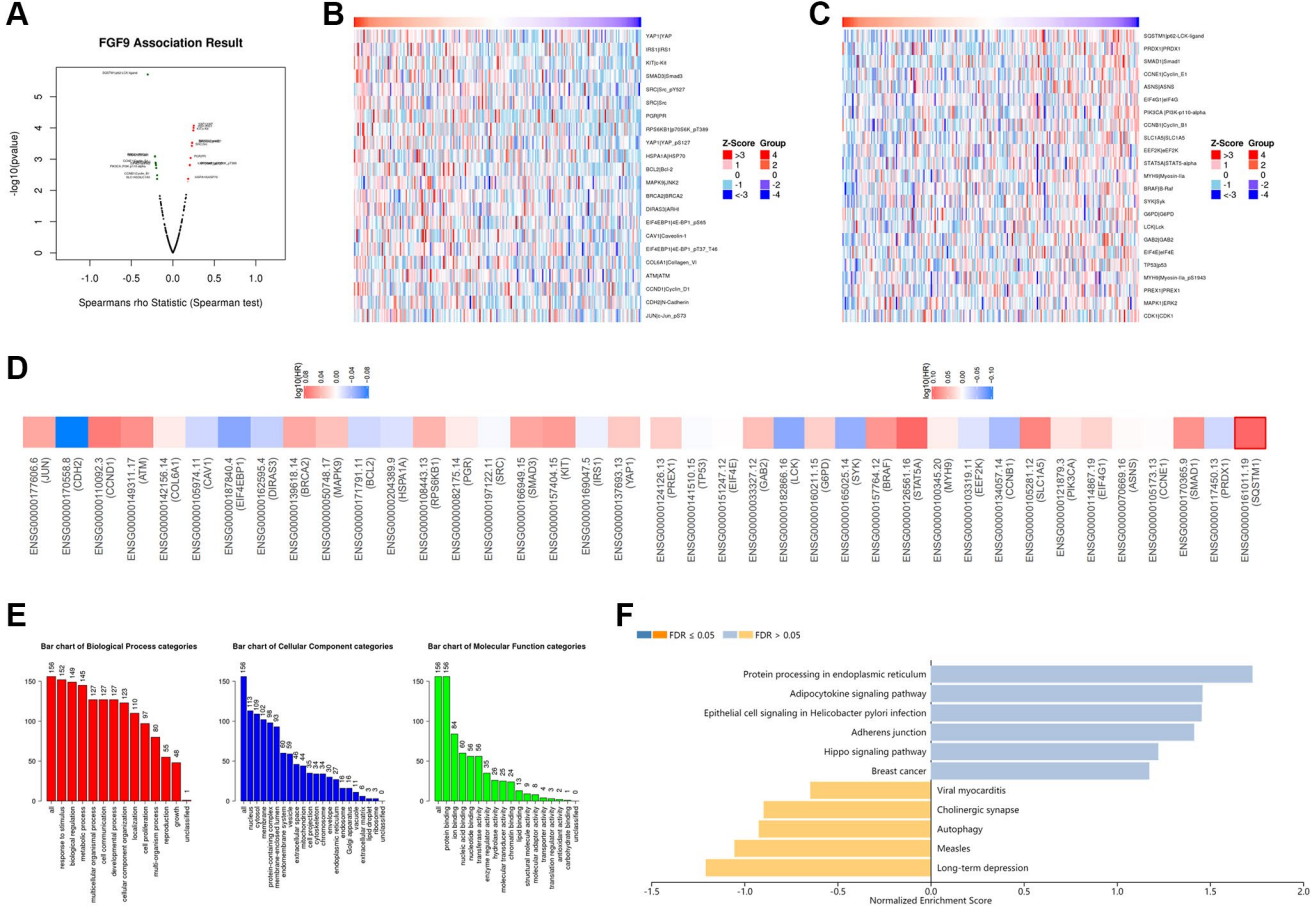
**The co-expression network of FGF9 in OC.** (**A**) The LinkedOmics platform portraying the crucially associated genes with FGF9 in OC patients. (**B**, **C**) Heatmaps showing the top genes that were positively and negatively correlated with FGF9 in OC. (**D**) Survival heatmaps downloaded from the GEPIA2.0 database displayed that the top genes that were positively and negatively associated with FGF9 in OC. (**E**, **F**) GO signaling pathway and KEGG signaling pathway of FGF9 in OC patients.

The LinkedOmics platform was applied to explore the co-expression network in the TCGAOV cohort, and then figuring out the biological functions of FGF9 in OC patients’ development. Figure 4A and Supplementary Table 2 have demonstrated the co-expressed genes that have positive and negative relationship with FGF9 (*p* < 0.05). Also, the heatmaps have depicted the genes that are positively and negatively correlated with FGF9 (Figure 4B, 4C and Supplementary Tables 3 and 4). Significantly, the top 19 positive-related genes have the great possibility to be low-risk molecules for OC patients. In addition, the top 20 negatively correlated genes that were high-risk molecules in OC. Meanwhile, 1 of top 20 negatively related genes owned the bad hazard ratio (Figure 4D). Moreover, through the investigation of Gene Ontology pathway, the result conveyed that the co-expressed genes of FGF9 participated in several biological process categories, such as response to stimulus, biological regulation and metabolic process. In the cellular component categories, these genes mainly take part in nucleus, cytosol and membrane. At the same time, the co-expressed genes of FGF9 significantly join in the protein binding, ion binding and nucleic acid binding in the molecular function categories (Figure 4E). Moreover, the KEGG analysis has implied that the enriched pathways were protein processing in endoplasmic reticulum, adipocytokine signaling pathway, epithelial cell signaling in helicobacter pylori infection, adherens junctions etc. (Figure 4F).

### The link between FGF9 with immune regulation

**Figure 5:**
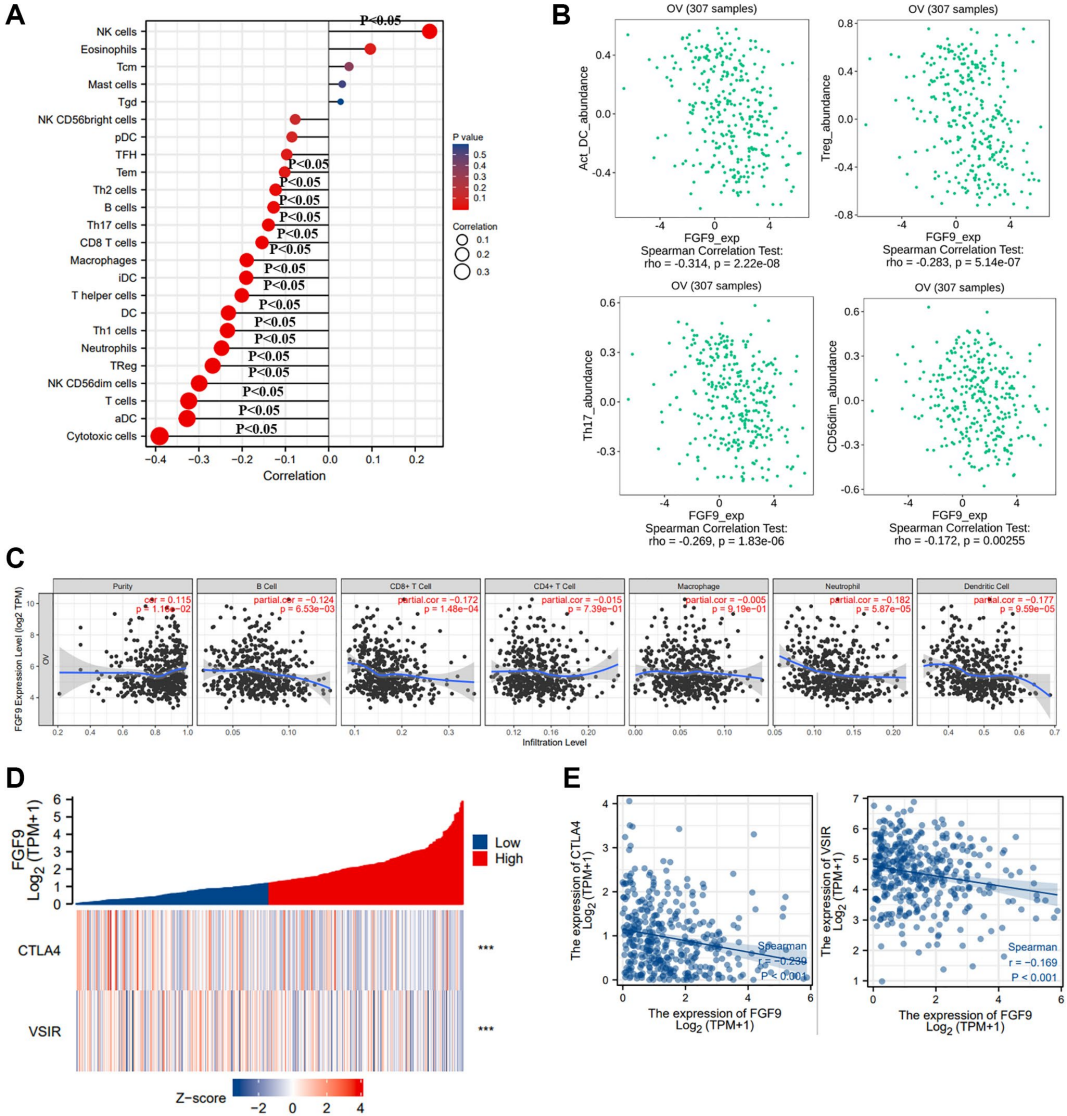
**The relationship between the expression level of FGF9 and immune responses of OC patients.** (**A**) The diagraph showing the relation between FGF9 expression and 24 types of immune cells. The size of the dots represented the values of Spearman r (*p* < 0.05). (**B**) The pictures downloaded from TISIDB database showing the relationship between FGF9 and immune infiltration cells, such as activated dendritic cells (aDC), Treg, Th17 cells, NK CD56dim cells (*p* < 0.05). (**C**) The Timer database showing the relationship between the expression level of FGF9 and immune infiltration cells. (**D**, **E**) The heatmap and scatterplot depicting FGF9 expression was negatively correlated to VSIR or CTLA4.

Then, we downloaded the statistics of TCGA-OV database and analyzed the effects of FGF9 expression on immune regulation of ovarian cancer patients via ssGSEA. It indicated that FGF9 was positively linked to the infiltration of natural killer (NK) cells (*p* < 0.05). Meanwhile, FGF9 expression was negatively correlated with cytotoxic cells, activated dendritic cells (aDC), T cells, NK CD56dim cells, Treg, neutrophils and T helper type 1 (Th1) cells (*p* < 0.05) (Figure 5A). In addition, the results that obtained from TISIDB database were consistent with the findings before (Figure 5B). By means of TIMER database, the plot further portrayed that the expression level of FGF9 had strong negative relationship with B cell, CD8^+^ T cell, CD4^+^ T cell, macrophage, neutrophil and dendritic cell (*p* < 0.05) (Figure 5C). Furthermore, the figures have depicted that the relationship between FGF9 expression and immune checkpoints and the findings showed that the expression level of FGF9 was negatively linked with CTLA4 (Spearman r = −0.239, *p* < 0.001) and VSIR (Spearman r = −0.169, – < 0.001) (Figure 5D, 5E).

Additionally, we further investigated the relation between FGF9 and immune responses through the TISIDB platform, including immunoinhibitors and receptors. This picture has conveyed the correlation between immunoinhibitors in OC and the expression of FGF9 (Supplementary Figure 1A). The findings implicated that the top four immunoinhibitors that were strongly linked with FGF9 were PDCD1LG2 (Spearman r = −0.331, *p* = 3.36e-09), HAVCR2 (Spearman r = −0.317, *p* = 1.66e-08), CD274 (Spearman r = −0.314, *p* = 2.19e-08) and LAG3 (Spearman r = −0.309, *p* = 3.96e-08) (Supplementary Figure 1B). Moreover, the correlation between FGF9 and receptors has been conducted (Supplementary Figure 2A). And the top four receptors that had negative association with FGF9 expression were CXCR6 (Spearman r = −0.289, *p* = 2.96e-07), CXCR3 (Spearman r = −0.271, *p* = 1.52e-06), CCR5 (Spearman r = −0.264, *p* = 3.04e-06) and CCR1 (Spearman r = −0.259, *p* = 4.7e-06) (Supplementary Figure 2B). In summary, these results have demonstrated that FGF9 was involved in the immune regulation of OC patients.

## DISCUSSION

The purpose of this paper was to figure out the correlation of exosome-associated genes and the progression in ovarian cancer. By exploring the co-DEGs between an exosome-associated gene dataset and two OC datasets obtained from the GEO database, we could find two up-regulated genes CD24 and CP. And we concluded the expression level of FGF9 displayed downregulation of OC. Additionally, the higher expression of FGF9 tended to be correlated with the better prognosis of OC patients. Consequently, the findings have concluded that the expression of FGF9 was different in normal ovarian tissues and OC tissues. FGF9 expression decreased in OC group. By means of LinkedOmics database, we could figure out the positively and negatively associated genes with FGF9 in OC patients. Meanwhile, FGF9 showed a correlation with the prognosis of OC patients. Also, the results of GO pathway and KEGG pathway conveyed that the co-expressed genes participated in microenvironment of cancer cells and the biological functions in treating cancers.

It has been reported that various kinds of cells could generate exosomes. And the diameters of exosomes are 30–150 nm. Moreover, exosomes have been identified to play an essential role in the cells’ communication via miRNA, mRNA, and DNA [25]. Recently, there is an increasing trend concerning the exosome nanotechnology [26]. Because exosome possessed the special structure and favorable biological characteristic, the studies regarding exosome could be beneficial to the nano medical therapy in the future [27]. Additionally, exosomes could be secreted by tumor cells and they could exert great effects in changing the microenvironment of tumors. At the same time, it was verified that exosomes were significant in the cancer progression and the antitumor immunity [28, 29]. Several signaling pathways could be induced and suppressed by exosomes. Moreover, exosomes could contribute to the metastasis and drug resistance of cancer cells [30]. Significantly, exosomes that were generated from OC cells could induce niche formatting before metastasis through inhibiting immune responses, angiogenesis and the reformation of oncogenes [31]. A recent study has indicated that, in OC cells, the exosomal miR-1246 induce the development of tumors. Besides, the inhibitors of miR-1246 could lead to the downregulated level of PDGFRβ and ki67 *in vivo* trials. Also, this paper has demonstrated that miR-1246 secretion through exosomes could elevate the chemoresistance in OC patients [32]. In addition, another study has illustrated that exosomal pGSN could elevate the survival of OC cells through the conversion of the chemosensitivity into chemoresistance in OC cells [33]. These results have implicated that in OC patients’ progression, exosome was importantly vital. And to further investigate the underlying mechanism of exosome and OC could benefit the future therapies for OC patients. Our study is aimed at exploring the prognostic value of FGF9 in OC patients and we concluded that high expression level of FGF9 was followed with good prognosis.

FGF9, as a target gene of miR-214, could suppress cancer-associated fibroblasts (CAFs) in GC cells. Also, the abnormal FGF9 expression in CAFs has strong relationship with poorer prognosis of GC patients [34]. A study implicated that HD5 expressed in ovarian cancer cells via western blot and immunohistochemistry (IHC) evaluations. And the expression of HD5 mediated by FGF9 signaling pathway further verified HD5 was correlated with cancer [35]. The analysis has conveyed that overexpression of FGF9 could provide a new clinical strategy in gastric cancer and bladder cancer. P4, as a new FGF9-binding peptide, could play a pivotal part in enhancing the sensitivity of chemical drugs [36]. Low expression of miR-187 was linked to diminished overall survival (OS) of cervical cancer patients. The over-expressed level of miR-187 suppressed cell growth and triggered cell apoptosis in cervical cancer [37]. FGF9 could act as a target gene of microRNA-219a-5p and relieved the chemoresistance of cisplatin in non-small cell lung cancer (NSCLC) [38]. In this paper, the exosome-related gene FGF9 expression was high in normal ovarian tissues compared to OC tissues.

Several studies have proven that the combination of immunotherapy and radiotherapy could provide a new strategy of therapy in ovarian cancer patients. MSI-H/dMMR and immune checkpoint inhibition could synergize with other strategies to exert effects in the treatment of ovarian cancer. Cancer vaccines and immunomodulation were reported to possess the potential to be low-toxicity approaches for OC patients [39–41]. In this article, we have explored the correlation between FGF9 and immune responses. FGF9 was reported to have a positive link with NK cells. On the other hand, FGF9 had a negative correlation with cytotoxic cells, aDC, T cells, NK CD56dim cells, Treg, neutrophils and Th1 cells. Simultaneously, FGF9 was crucially related to immunoinhibitors (PDCD1LG2, HAVCR2, CD274, LAG3) and receptors (CXCR6, CXCR3, CCR5, CCR1). Moreover, cancers might evade from the attack of NK cells. Interestingly, to reinforce the cytotoxicity of NK cells could elevate the efficacy of NK-associated immunotherapy [42]. Also, in high-grade serous ovarian cancer (HGSC), NK cells could be pivotal in the anticancer immunity triggered by immune checkpoint blockade [43]. This study also concluded that FGF9 expression had a negative relation with immune checkpoints, including CTLA4 and VSIR. CTLA4 belonged to the family of Immunoglobulin-associated receptors and it was expressed via CD4^+^ and CD8^+^ T cells. Through this way, CTLA4 could take part in activating the T cells and induce immune regulation [44]. Nowadays, immune checkpoint inhibitors were applied for the therapy of ovarian cancer. CTLA4 could exert great effects in eliminating autoreactive T cells and PD-1 controlled T cells’ apoptosis [45]. These above results have implicated that FGF9 was correlated with immune responses and regulation, implying that FGF9 could be a promising biomarker for the immunotherapy of OC patients.

## CONCLUSIONS

In conclusion, this paper has reported that the expression level of FGF9 had a vital relationship with the prognostic prediction value of OC. Furthermore, it has been identified that FGF9 was crucially related with the immunoinhibitors and chemokine receptors. Consequently, this study has provided a novel horizon that the exosome-correlated gene FGF9 had the great potential to be a prognostic prediction strategy for OC patients.

## Supplementary Materials

Supplementary Figures

Supplementary Table 1

Supplementary Table 2

Supplementary Tables 3 and 4
