# Supplementary material for: Downregulated exosome-associated gene FGF9 as a novel diagnostic and prognostic target for ovarian cancer and its underlying roles in immune regulation
Source: Aging (Albany NY). 2022 Feb 21;14(4):1822–35. doi: 10.18632/aging.203905 (PMC8908935; doi:10.18632/aging.203905)
Supplement: Supplementary Figures [file aging-14-203905-s001.pdf]

SUPPLEMENTARY FIGURES

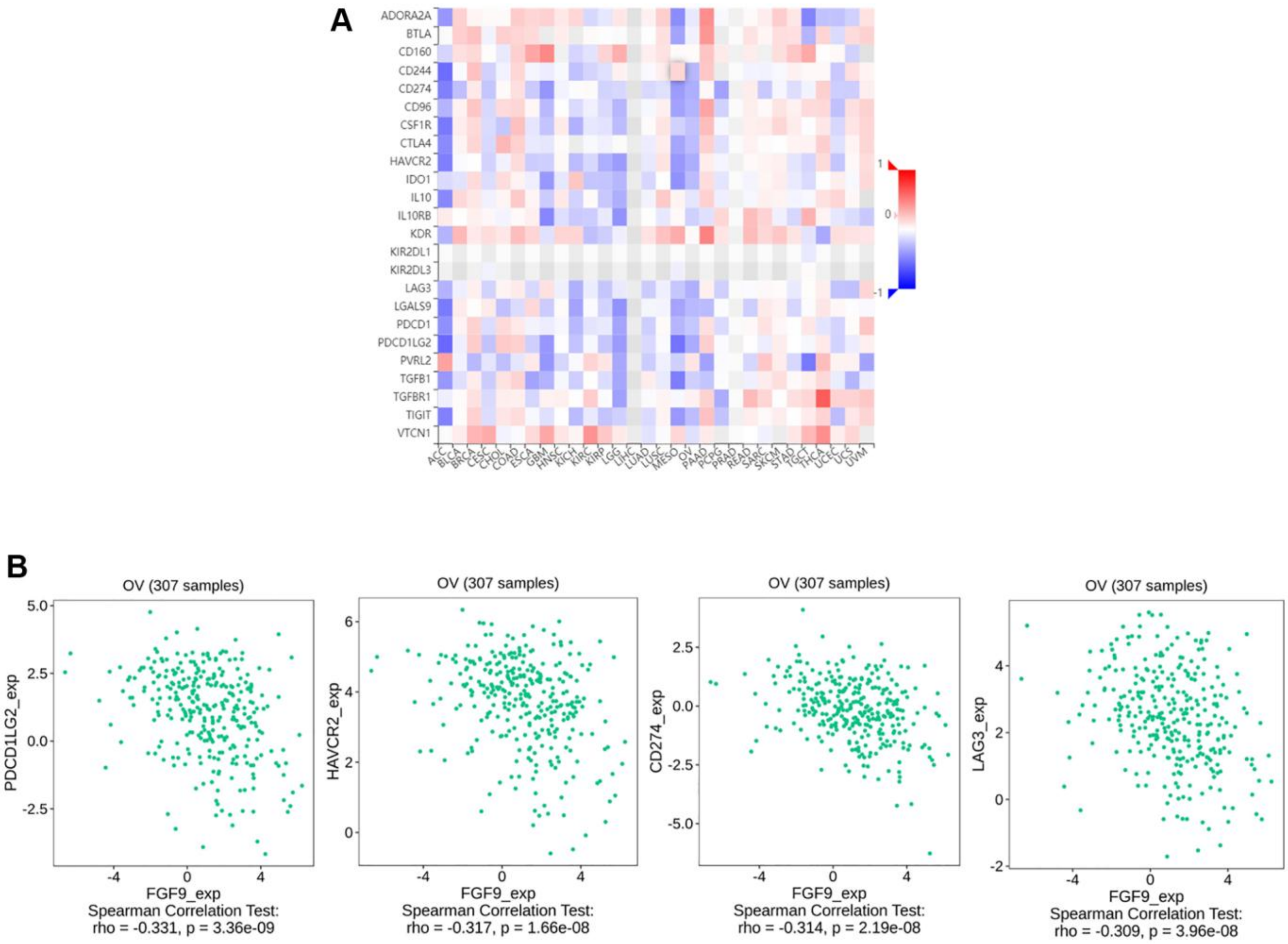

Supplementary Figure 1. The relationship between the expression level of FGF9 and immunoinhibitors of OC patients. (**A**) The diagraph showing the correlation between FGF9 expression and immunoinhibitors. (**B**) The scatter plots depicting the top four immunoinhibitors sharing a negative relationship with FGF9 expression.

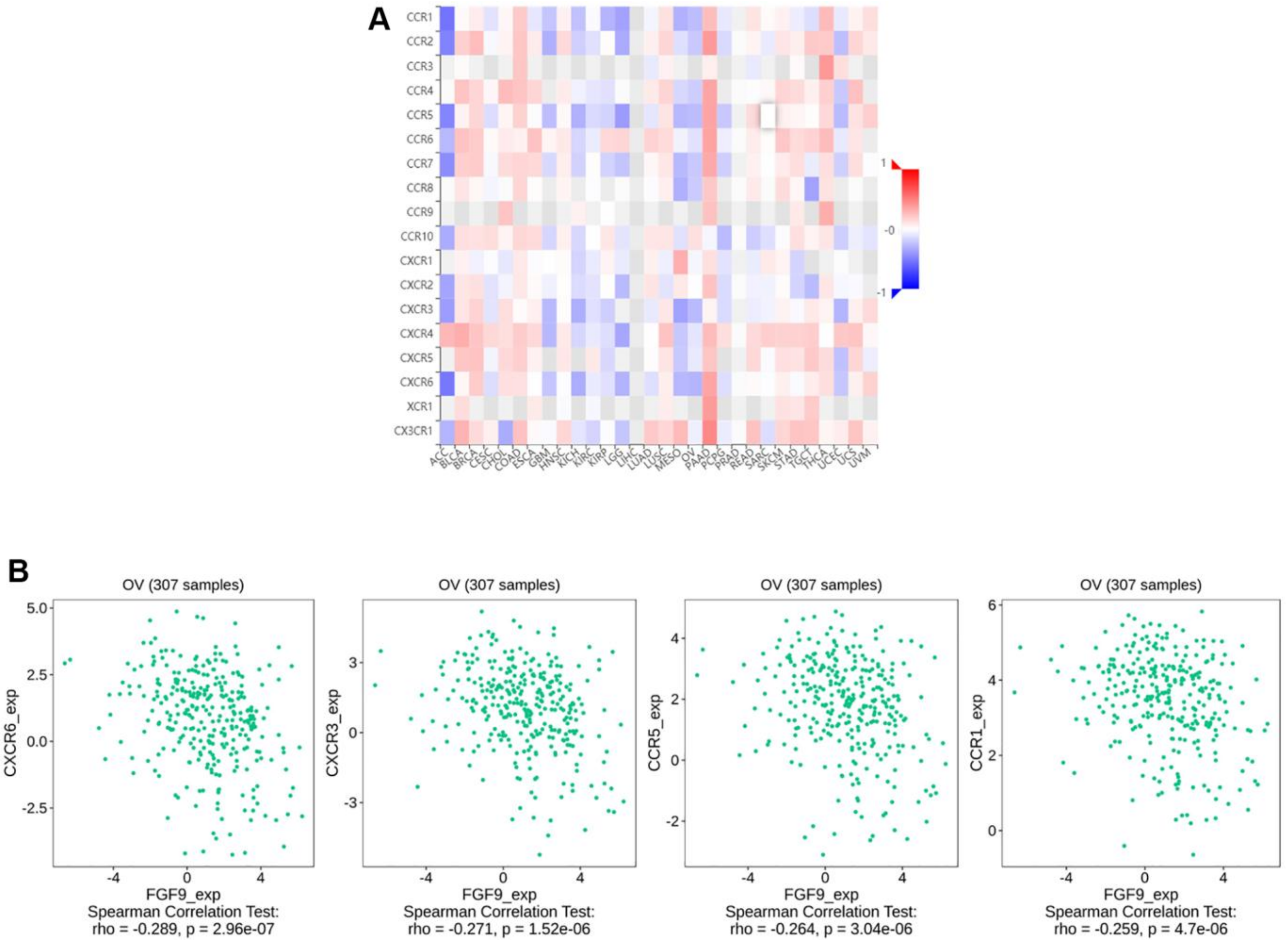

Supplementary Figure 2. The correlation between the FGF9 expression and chemokine receptors of OC patients. (**A**) The picture showing the connection between FGF9 expression and receptors. (**B**) The scatter plots portraying the top four receptors that possessed a negative relationship with FGF9 expression.
