# Supplementary material for: Downregulated exosome-associated gene FGF9 as a novel diagnostic and prognostic target for ovarian cancer and its underlying roles in immune regulation
Source: Aging (Albany NY). 2022 Feb 21;14(4):1822–35. doi: 10.18632/aging.203905 (PMC8908935; doi:10.18632/aging.203905)
Supplement: Supplementary Tables 3 and 4 [file aging-14-203905-s004.pdf]

SUPPLEMENTARY TABLES

Supplementary Table 3. The top 19 genes positively correlated with FGF9 in ovarian cancer.

YAP1	PGR	BRCA2	ATM
IRS1	RPS6KB1	DIRAS3	CCND1
KIT	HSPA1A	EIF4EBP1	CDH2
SMAD3	BCL2	CAV1	JUN
SRC	MAPK9	COL6A1	

Supplementary Table 4. The top 20 genes negatively correlated with FGF9 in ovarian cancer.

SQSTM1	EIF4G1	MYH9	LCK
PRDX1	PIK3CA	STAT5A	GAB2
SMAD1	SLC1A5	BRAF	EIF4E
CCNE1	CCNB1	SYK	TP53
ASNS	EEF2K	G6PD	PREX1
